# Childhood trauma is associated with perceived stress and hair cortisol levels characterized by the BDNF Val66Met genotype and sex

**DOI:** 10.3389/fpsyt.2025.1526791

**Published:** 2025-05-21

**Authors:** Zhenxu Li, Se Jun Koo, Hye Yoon Park, Jee Eun Min, Jung Tak Park, Eun Lee, Suk Kyoon An

**Affiliations:** ^1^ Section of Self, Affect and Neuroscience, Institute of Behavioral Science in Medicine, Yonsei University College of Medicine, Seoul, Republic of Korea; ^2^ Yonsei University College of Medicine, Seoul, Republic of Korea; ^3^ Institute of Kidney Disease Research, Yonsei University College of Medicine, Seoul, Republic of Korea; ^4^ Department of Internal Medicine, Yonsei University College of Medicine, Seoul, Republic of Korea; ^5^ Department of Psychiatry, Yonsei University College of Medicine, Severance Hospital, Seoul, Republic of Korea

**Keywords:** BDNF, Val66Met, childhood trauma, perceived stress, hair cortisol concentration

## Abstract

**Introduction:**

Childhood trauma increases the risk of mental disorders by affecting both psychological and physiological stress responses in adulthood, including perceived stress and long-term hypothalamic-pituitary-adrenal (HPA) axis activity. The mechanisms underlying these effects may involve gene-environment (G × E) interactions, with the brain-derived neurotrophic factor (BDNF) gene Val66Met polymorphism and sex playing important roles. This study aims to investigate how childhood trauma influences stress responses, considering the BDNF Val66Met polymorphism and sex differences.

**Methods:**

Secondary data from 190 healthy young adults (96 female) were analyzed. Childhood trauma and perceived stress were assessed using the Childhood Trauma Questionnaire (CTQ) and the Perceived Stress Scale (PSS), respectively, and hair cortisol concentration (HCC) was assessed as a measure of long-term cortisol levels. Participants were genotyped for the BDNF Val66Met polymorphism and stratified as Val/Val or Met carriers. Hierarchical linear regression models were used to examine the interactions between CTQ scores, BDNF Val66Met genotype, and sex to assess their effects on PSS scores and HCC. Additional analyses included separate linear regression models of the CTQ scores for HCC in Val/Val and Met carriers according to sex.

**Results:**

Higher CTQ scores were positively associated with PSS scores in the entire sample (B= 0.124, p = 0.002). No significant main effects of CTQ scores, BDNF Val66Met genotype, or sex on HCC were observed. However, a significant three-way interaction between CTQ scores, BDNF Val66Met, and sex on HCC was observed, with a positive association between HCC and childhood trauma observed exclusively in female Val/Val carriers (B= 0.019, p=0.034).

**Conclusion:**

These results demonstrated that childhood trauma elevates perceived stress across all participants and increases HCC levels, specifically in female Val/Val carriers.

## 1 Introduction

Childhood trauma, including physical, emotional, and sexual abuse, as well as physical and emotional neglect (1), is strongly associated with increased vulnerability to mental disorders, such as depression (2), schizophrenia (3), and post-traumatic stress disorder (PTSD) (4). The underlying pathways are unclear and may involve altered psychological and physiological stress responses, with childhood trauma considered an environmental stressor (5, 6).

Psychological responses to stress play a critical role in the development of mental illness (7, 8) and these responses can be influenced by childhood trauma. According to the stress sensitization framework (9), childhood trauma has long-term impacts on psychological sensitivity to stress. Cumulative advantage/disadvantage theory (10) suggests that individuals with childhood trauma may encounter a series of disadvantages later in life, indicating that stress can accumulate over time. Combined with numerous studies supporting the connection between childhood trauma and increased perceived stress in adulthood (11, 12), individuals with childhood trauma tend to perceive stress as overwhelming and adopt negative coping strategies (13), leading to a cycle of increased stress and heightened stress perception over time. Therefore, childhood trauma profoundly affects psychological stress responses such as perceived stress, contributing to mental disorders (9, 14).

In addition to its psychological impact, childhood trauma affects the activity of the hypothalamic-pituitary-adrenal (HPA) axis and alters the levels of cortisol, a stress hormone released during HPA axis activation (15). Childhood trauma often occurs during sensitive developmental periods, such as adolescence, when cortisol levels naturally increase and the HPA axis is particularly sensitive to stress (16, 17). Therefore, exposure to childhood trauma can overwhelm the developing stress-response system, leading to long-term dysregulation of HPA axis activity that may persist into adulthood, increasing vulnerability to chronic stress and the risk of mental disorders (17, 18).

Hair cortisol concentration (HCC) is used to measure long-term cortisol levels (12, 19, 20). In contrast to cortisol measurements from the serum, saliva, or urine (21), which can be affected by short-term fluctuations in cortisol secretion (22), HCC provides a time-averaged assessment over several months as cortisol accumulates in growing hair (23). Thus, HCC is a reliable indicator of long-term HPA axis activity and is useful for investigating the influence of childhood trauma on physiological stress response. However, previous studies have reported inconsistent results. One study found an association between childhood trauma and lower HCC (24), while others found no association (12, 25). Meanwhile, one study observed a sex-specific effect, showing elevated HCC levels in women with greater childhood trauma exposure (26). These inconsistencies suggest that other factors such as sex differences and genetic predisposition (27, 28) may influence the relationship between childhood trauma and HCC.

Gene-environment (G × E) interactions suggest that childhood trauma, as an environmental factor, can interact with genetic factors to influence biological mechanisms (29, 30) and the development of mental disorders (31, 32). Among the candidate genes sensitive to psychological stress, the brain-derived neurotrophic factor (BDNF) gene influences the HPA axis through the production of BDNF (33). The BDNF protein promotes the survival, growth, and maintenance of neurons (33, 34), which are crucial for brain regions such as the hippocampus and prefrontal cortex (35), both of which are involved in regulating the HPA axis (36, 37). Moreover, BDNF helps protect hippocampal neurons from damage caused by elevated cortisol (38). The BDNF Val66Met (rs6265) polymorphism is the most widely studied single nucleotide polymorphism (SNP) of the BDNF gene, leading to an amino acid substitution in which valine (Val) is replaced by methionine (Met) at codon 66 (39), with the Met allele exhibiting less BDNF gene activity than Val homozygotes (39, 40). The BDNF Val66Met polymorphism is linked to HPA axis activity. Specifically, research has shown that the Met allele is associated with higher cortisol levels than the Val/Val allele in people with depression (41), indicating a heightened stress response to acute stress. Furthermore, childhood trauma interacts with the BDNF Val66Met polymorphism to further shape the stress response. For example, a study of veterans with PTSD (29) found that childhood trauma interacts with BDNF Val66Met, particularly in Met carriers, leading to a blunted psychophysiological response to acute stress. In sum, these findings suggest that the BDNF Val66Met polymorphism, along with its interaction with childhood trauma, modulates stress responses, particularly in the context of acute HPA axis activity. However, the effects of childhood trauma × BDNF Val66Met interactions on long-term HPA axis activity, such as in HCC, remain unclear, although it has been suggested that Met carriers with childhood trauma may experience reduced hippocampal volume (42–44), potentially leading to prolonged HPA activation (36) and higher allostatic load (45) over time, which may result in elevated HCC levels (46).

Notably, sex may play a crucial role in shaping the interactions between childhood trauma, the BDNF Val66Met polymorphism, and physiological stress responses. First, sex differences exist in childhood trauma exposure, with strong evidence showing that females often report higher levels of childhood trauma than males (29, 47). Second, sex differences were observed in HCC levels, although the findings were mixed. Some studies have shown that males have higher HCC levels (2, 48), while another study reported higher HCC levels in girls (49); others have found no link with sex (46, 50). Third, sex differences also affect how the BDNF Val66Met variant influences HPA-axis activity, with most studies focusing on salivary cortisol responses to acute psychological stress and showing mixed results (26, 51, 52). Finally, sex may influence the interaction between early life stress and BDNF Val66Met on HCC levels. For example, one study found that higher neonatal pain-related stress predicted lower HCC levels in boys carrying the Met allele (28). However, few studies have examined the interactive effects of childhood trauma × BDNF Val66Met × sex on long-term cortisol levels in adults, leaving a gap in our understanding of these complex interactions.

To address this gap, this study examined the relationship between childhood trauma and psychological and physiological responses to stress in healthy young adults. Regarding psychological responses, the association between childhood trauma and perceived stress was investigated, with the expectation that childhood trauma would be associated with higher levels of perceived stress. Given the limited evidence on how BDNF Val66Met and sex influence the relationship between childhood trauma and perceived stress, the interaction effects were examined on an exploratory basis. For physiological responses, the relationship between childhood trauma and HCC was examined based on the hypothesis that childhood trauma is linked to elevated HCC levels. The study also investigated the interaction between childhood trauma and the BDNF Val66Met polymorphism and expected that Met carriers would show significantly altered HCC levels in response to childhood trauma compared with Val/Val carriers. Additionally, the hypothesis was extended to a three-way interaction between childhood trauma, BDNF Val66Met, and sex, suggesting that sex further modulates the impact of childhood trauma on HCC levels.

## 2 Methods

### 2.1 Study sample

Secondary data were obtained from a previously published cross-sectional study (53) conducted from November 2016 to July 2018. This study involved 191 Korean participants aged 19–30, recruited through online advertisements and recruitment posters. One participant was excluded due to missing test data, resulting in a final sample size of 190 participants. Given that psychiatric illness is associated with childhood trauma (3), perceived stress (54, 55), and cortisol levels (55), and thus may mask the specific effects of childhood trauma, BDNF Val66Met genotype, and sex, only healthy participants were included. All participants were screened for psychiatric illnesses using the Structured Clinical Interview for DSM-IV Axis I Disorders (SCID-IV). Exclusion criteria were as follows: 1) past or current psychiatric disorder, 2) lifetime neurological disorder, 3) head trauma history accompanied by loss of consciousness, 4) a medical or surgical condition requiring hospitalization, 5) hospital discharge in the past 6 months, 6) taking oral contraceptives and glucocorticoid medication, and 7) currently pregnant or breastfeeding. Written informed consent was obtained from all participants using procedures approved by the Institutional Review Board (IRB) of the Severance Hospital of the Yonsei University Health System (IRB No.2014-1767-035).

### 2.2 The Childhood Trauma Questionnaire (CTQ)

The CTQ (56) is a widely used screening tool for detecting childhood trauma, including neglect and abuse (57). It includes five subscales that evaluate different aspects of childhood trauma: emotional abuse, physical abuse, sexual abuse, emotional neglect, and physical neglect. CTQ scores can be interpreted by calculating the total score, with higher values reflecting greater levels of childhood trauma (58). This approach is widely accepted and commonly used in research (57). In this study, the CTQ demonstrated an internal consistency of 0.717, consistent with prior psychometric validation (58), supporting the robustness of CTQ in measuring trauma-related psychological stress despite its subjective nature.

### 2.3 The Perceived Stress Scale (PSS)

The PSS (59) is the most widely used psychological tool for evaluating an individual’s perception of stress over the past month. Higher PSS scores indicate greater levels of negative distress and poorer positive coping abilities (60). The ten-item Korean version of the PSS (61) has demonstrated reliability and validity (score range: 10–50) and was therefore used in this study. The internal consistency of the PSS in this study was 0.789, reinforcing the robustness of PSS in measuring perceived psychological stress despite its subjective nature.

### 2.4 Hair cortisol analysis

Hair cortisol levels were measured following the procedures detailed in the primary study (53). In brief, 10 strands/10 mg of hair were collected from each participant and cut near the scalp, with a 3 cm segment used to estimate cortisol concentration over the past three months (62). Cortisol levels were quantified via enzyme-linked immunosorbent assay (ELISA), expressed as the cortisol-to-protein ratio (pg hair cortisol/μg hair protein). For further methodological details, please refer to the primary study (53).

### 2.5 Val66Met genotyping

Genotyping of Val66Met (rs6265) was performed by Macrogen, Inc. using standard PCR amplification and Sanger sequencing methods, as described in the primary study (53). Based on the results of the analysis, genotype carriers were divided into two groups: Met allele carriers and Val/Val carriers. Full genotyping procedures are available in the primary publication (53).

### 2.6 Statistical analysis

Statistical analyses were performed using SPSS version 25 (IBM, Chicago, IL, USA) and R software (https://www.r-project.org/). Participants were divided into two BDNF Val66Met genotype groups (Val/Val and Met carriers) and tested for demographic characteristics. Bivariate associations among the study variables were examined using Pearson’s correlation and t-tests. To approximate a normal distribution, HCC levels were log-transformed, and the BDNF Val66Met genotypes and sex were transformed into dummy variables for further analysis. The CTQ scores were calculated as the total score to reflect overall trauma exposure.

A hierarchical linear regression model was used to examine the association between PSS and CTQ score and further explore the potential effects of the Val66Met genotype and sex. In this model, Step 1 included the main effects of CTQ score, the BDNF Val66Met genotype, and sex on PSS score. In Step 2, the interaction term between CTQ score and the BDNF Val66Met genotype was added, and in Step 3, a three-way interaction term involving CTQ score, the BDNF Val66Met genotype, and sex was included.

A similar hierarchical linear regression model was applied to assess the relationship between HCC and CTQ score, along with the interaction effects of BDNF Val66Met and sex. Step 1 included the main effects of CTQ scores, the BDNF Val66Met genotype, and sex on HCC. Step 2 added the interaction term for CTQ score and the BDNF Val66Met genotype, whereas Step 3 included the three-way interaction of CTQ score, the BDNF Val66Met genotype, and sex. To further investigate the associations between CTQ score and HCC within specific groups, separate linear regression analyses were conducted for four subgroups: female Val/Val, female Met, male Val/Val, and male Met carriers. A two-tailed significance level of 0.05 was applied to determine statistical significance.

## 3 Results

### 3.1 Study participants

The demographic characteristics, CTQ and PSS scores, and HCC levels of the 190 participants are presented in **Table 1**. Data are provided for the total sample and stratified by Val66Met genotype (Val/Val carriers versus Met carriers) and sex. There were no significant differences in sex distribution, CTQ scores, PSS scores, or HCC levels between the BDNF Val/Val and Met carriers. The BDNF Val66Met genotype distribution did not differ significantly from Hardy–Weinberg expectations (χ2 = 0.14, *p* = 0.93). Sex differences were observed in terms of age (*p* = 0.036) and years of education (*p* = 0.043), with men being older and having fewer years of education, likely due to mandatory military service requirements. To address potential confounding effects, however, sensitivity analyses were performed with age and education included as covariates, confirming the robustness of the findings, with further details provided in the following subsections. In addition, there was no significant relation between HCC and PSS (p = 0.829).

**Table 1:** Column heading: Demographic characteristics and CTQ, PSS score by Val66Met genotype and by sex.

Variable	Total (n= 190)	Val/Val (n= 59)	Val/Met or Met/Met (n= 131)	*p*	Female (n=96)	Male (n=94)	*p*
Age, mean (SD), years	23.0 (2.6)	23.0 (2.5)	23.0 (2.6)	0.873	22.6 (2.6)	23.4 (2.6)	0.036
Sex, N(%)				0.074			
Female	96 (50.5)	36 (61.0)	60 (45.8)				
Male	94 (49.5)	23 (39.0)	71 (54.2)				
Education, mean (SD), years	14.4 (1.4)	14.3 (1.4)	14.4 (1.4)	0.702	14.6 (1.5)	14.2 (1.3)	0.043
CTQ, mean (SD)	37.6 (10.1)	37.7 (9.9)	37.6 (10.3)	0.939	37.2 (9.8)	38.0 (10.5)	0.575
PSS, mean (SD)	25.7 (5.5)	26.2 (5.6)	25.4 (5.4)	0.366	26.0 (5.4)	25.3 (5.6)	0.379
HCC, median(IQR), pg /μg	5321.8 (6014.9)	5399.8 (6870.3)	5112.7 (5602.7)	0.243	8923.8 (6039.1)	5768.6 (5943.8)	0.061

Sex and BDNF Val66Met genotypes were entered as dummy variables (male=0, female=1; Val/Val carriers=0, Met carriers=1). CTQ, the Childhood Trauma Questionnaire; PSS, the Perceived Stress Test; HCC, hair concentration.

### 3.2 Associations of childhood trauma with perceived stress

**Figure 1:**
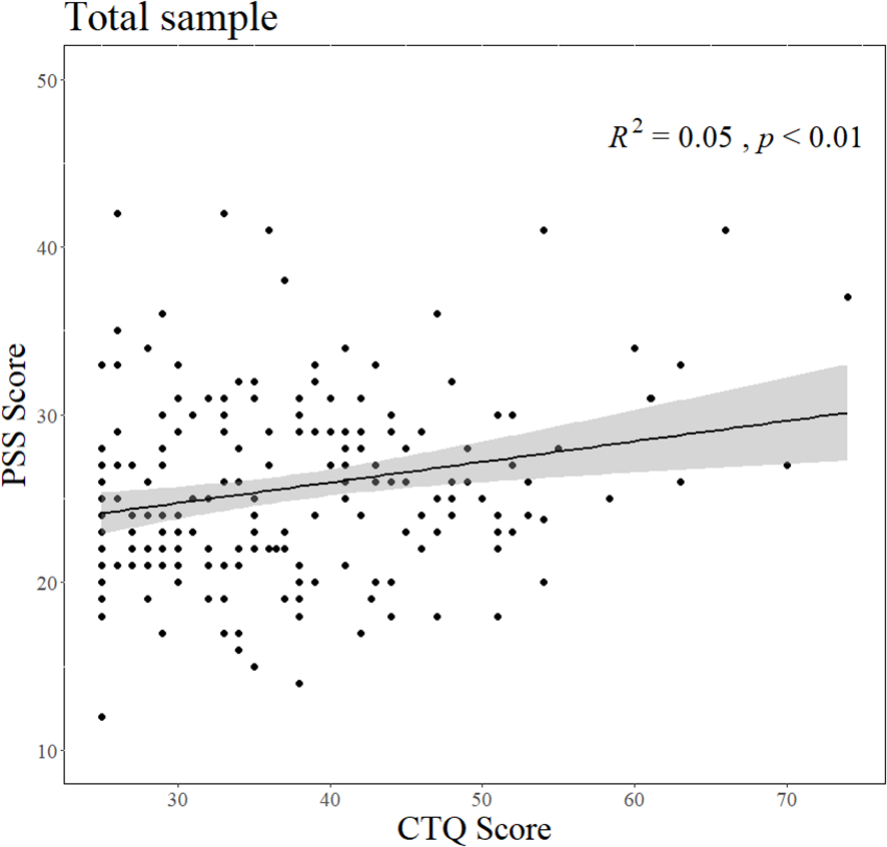
Association of CTQ score with PSS score in total sample. The line in the scatter plot represents the simple linear regression line. The shaded area indicates the 95% confidence interval. R^2^ value represents the strength of this relationship. CTQ, the Childhood Trauma Questionnaire; PSS, the Perceived Stress Scale.

Hierarchical linear regression analysis showed that PSS scores were positively related to CTQ scores (B = 0.124, *p* = 0.002; Step 1). A sensitivity analysis controlling for age and years of education confirmed the robustness of this association, though with a slightly altered effect size (B = 0.234, p = 0.017). For exploratory purposes, there were no interactions of CTQ score × BDNF Val66Met (*p* = 0.712; Step 2) or CTQ score × BDNF Val66Met × sex (*p* = 0.081; Step 3) with PSS scores. The detailed regression model results are presented in **Table 2**, and the association between the CTQ and PSS scores is illustrated in **Figure 1**.

**Table 2:** Column heading: Hierarchical linear regression model of PSS score with CTQ score, Val66Met genotype, and sex.

PSS Score				
Step	Variable	B	95% CI	*p*
Step 1	CTQ Score	0.124	[0.048, 0.200]	0.002
	BDNF Val66Met	-0.670	[-2.349, 1.008]	0.431
	Sex	0.720	[-0.835, 2.275]	0.362
Step 2	CTQ Score × BDNF Val66Met	-0.032	[-0.200, 0.137]	0.712
Step 3	CTQScore × BDNFVal66Met × Sex	0.302	[-0.038, 0.640]	0.081

Sex and BDNF Val66Met genotypes were entered as dummy variables (male=0, female=1; Val/Val carriers=0, Met carriers=1). CI, confidence interval, CTQ, the Childhood Trauma Questionnaire; PSS, the Perceived Stress Scale.

### 3.3 Associations of childhood trauma, BDNF Val66Met genotype, and sex with HCC

The results of the hierarchical linear regression model are presented in **Table 3**. In Step 1, no significant main effect of CTQ score on HCC was found in the total sample (p= 0.452). Similarly, there were no significant associations between HCC and the BDNF Val66Met genotype (p = 0.626) or sex (p = 0.075). In Step 2, the interaction effect of CTQ score and the BDNF Val66Met genotype on HCC was not significant (p = 0.593). In Step 3, the three-way interaction among CTQ score, the BDNF Val66Met genotype, and sex showed a significant association with HCC (p = 0.014), although the overall model did not reach statistical significance (F_7,182_ = 1.859, p=0.079). A sensitivity analysis controlling for age and years of education confirmed the robustness of this interaction (p = 0.015), with the overall model remaining non-significant (F_9, 180_ = 1.434, p = 0.176).

**Table 3:** Column heading: Hierarchical linear regression model of HCC with CTQ score, Val66Met genotype, and sex.

HCC level				
Step	Variable	B	95% CI	*p*
Step 1	CTQ Score	0.002	[-0.003, 0.007]	0.452
	BDNF Val66Met	-0.028	[-0.141, 0.085]	0.626
	Sex	0.095	[-0.010, 0.199]	0.075
Step 2	CTQ Score × BDNF Val66Met	-0.003	[-0.014, 0.008]	0.593
Step 3	CTQ Score × BDNF Val66Met × Sex	-0.028	[-0.051, -0.006]	0.014

Sex and BDNF Val66Met genotypes were entered as dummy variables in total sample analysis. CI, confidence interval; CTQ, the Childhood Trauma Questionnaire; HCC hair cortisol concentration.

**Figure 2:**
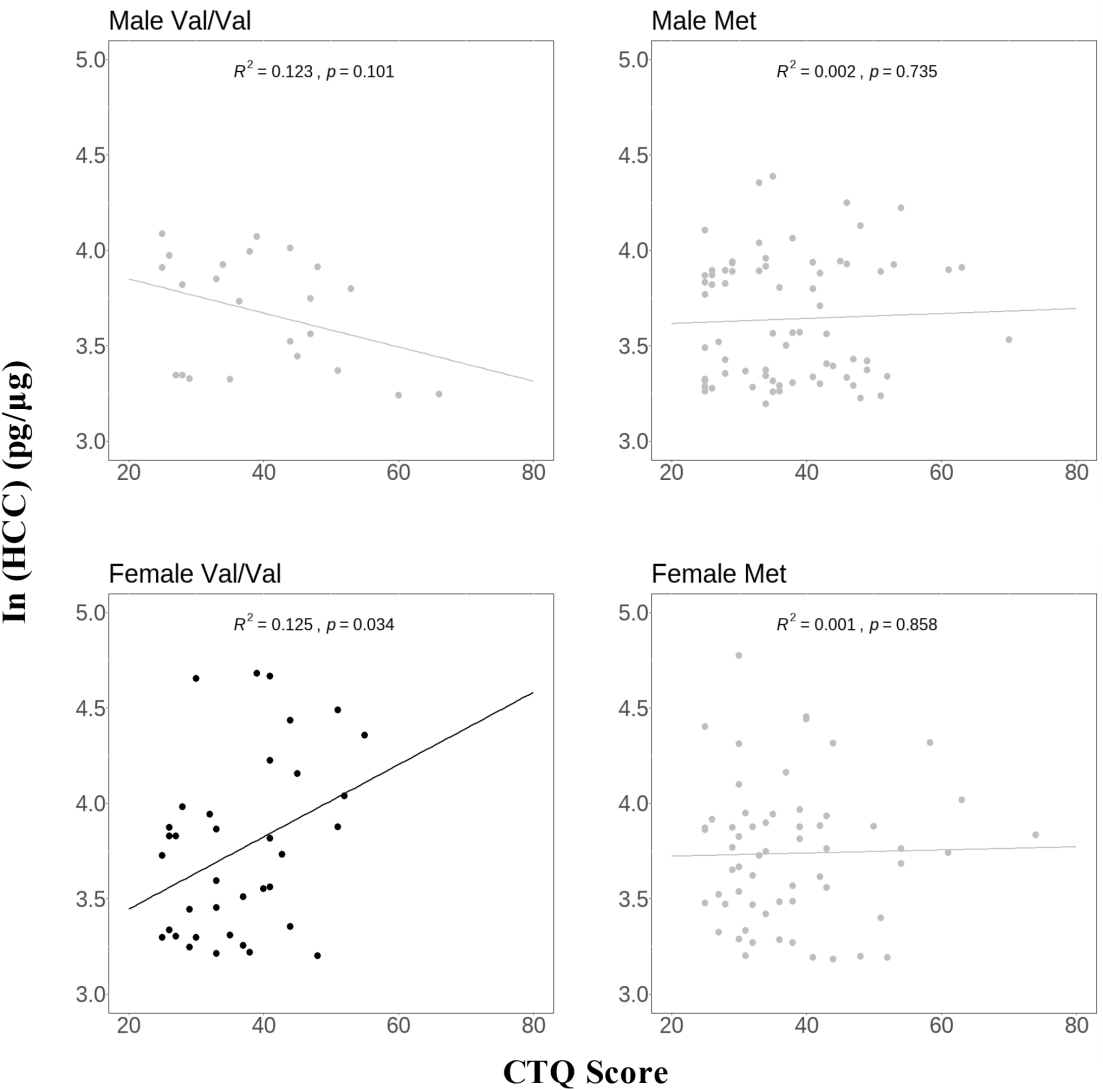
Association of HCC with CTQ score in groups divided by sex and Val66Met genotype. Y‐axis, hair cortisol values calculated by cortisol‐to‐protein ratio (pg hair cortisol/μg hair protein) and then transformed into natural log values, X‐axis, CTQ score; the line in the scatter plot represents the simple linear regression line. R^2^ value represents the strength of this relationship. CTQ, the Childhood Trauma Questionnaire; HCC, hair cortisol concentration.

To further investigate sex and Val66Met genotype differences, the sample was divided into four groups: male Val/Val carriers (n = 23), male Met carriers (n = 71), female Val/Val carriers (n = 36), and female Met carriers (n = 60). As shown in **Figure 2**, a positive association between HCC and CTQ score was observed exclusively in female Val/Val carriers (B=0.019, p=0.034), with no significant association in the other three groups (all p > 0.101).

## 4 Discussion

This study examined the relationship between childhood trauma and psychological and physiological stress responses among healthy young adults, considering the effects of the BDNF Val66Met genotype and sex differences. We hypothesized that childhood trauma increases perceived stress (Hypothesis 1), elevates HCC (Hypothesis 2), interacts with BDNF Val66Met to affect HCC (Hypothesis 3), and interacts with both BDNF Val66Met and sex to influence HCC (Hypothesis 4). Our findings fully supported Hypothesis 1, with higher childhood trauma exposure significantly associated with increased PSS scores across all participants, regardless of sex or BDNF Val66Met genotype. Our findings did not support Hypothesis 2, as no significant main effect of childhood trauma on HCC was observed in the total sample. Our findings partially supported Hypothesis 3, with no significant two-way interaction between childhood trauma and BDNF Val66Met observed, though a significant three-way interaction emerged. Our findings fully supported Hypothesis 4, as the three-way interaction of childhood trauma × BDNF Val66Met × sex was significantly associated with HCC, specifically revealing elevated HCC in female Val/Val carriers with greater childhood trauma exposure, but not in males of either genotype or in females with the Met allele. Overall, childhood trauma significantly influenced psychological responses and the interaction of childhood trauma × BDNF Val66Met × sex influenced physiological responses. Notably, a strong effect was observed in females with the Val/Val genotype, highlighting the importance of these interactions in long-term stress regulation.

Regarding perceived stress, childhood trauma was positively associated with perceived stress across all participants. This finding aligns with previous research (12, 26, 63, 64), highlighting the strong relationship between childhood trauma and stress perception. According to the PSS questionnaire (51, 61), high levels of perceived stress reflect increased negative distress and poor coping abilities. This suggests that individuals with greater childhood trauma may not only feel overwhelmed by stress but also have more difficulty coping effectively, which may result in more psychological stress. Given that elevated levels of perceived stress are associated with various mental health conditions such as depression (65) and anxiety (8), childhood trauma may contribute to the development of psychopathology by increasing perceived stress. In addition, no significant interaction between childhood trauma and the BDNF Val66Met genotype or a three-way interaction with sex was observed for perceived stress, suggesting that childhood trauma independently contributes to perceived stress without interacting with the Val66Met genotype and sex.

Regarding HCC in relation to childhood trauma, there was no main association between childhood trauma and HCC. This result aligns with several previous studies showing non-significant results (12, 25, 66), while other studies have reported significant associations (24, 26). This suggests that factors such as BDNF Val66Met genotype and sex, as examined in this study, may contribute to the complex relationship between childhood trauma and HCC.

Regarding HCC in relation to the two-way interaction of childhood trauma with the BDNF Val66Met genotype, no significant interaction effect on long-term HPA axis activity was found. However, this result contradicts our hypothesis that Met carriers exposed to childhood trauma have higher HCC levels. The non-significant results of this study suggest that more complex mechanisms may underlie this relationship, potentially influenced by other factors such as sex. Sex differences, in particular, may dilute the impact of this interaction on physiological stress responses, as this study observed a complicated three-way interaction involving sex.

Regarding HCC in relation to the three-way interaction of sex, childhood trauma, and the BDNF Val66Met genotype, a positive association between HCC and childhood trauma was found exclusively in women with the Val/Val genotype. This suggests that women with the Val/Val genotype may experience the most pronounced impact of childhood trauma on long-term cortisol response, compared to other sex and genotype groups.

A possible explanation may lie in the combined interactions of sex with the BDNF Val66Met genotype and sex with childhood trauma, which together may influence HPA reactivity to acute stress and relate to HCC levels (67). In terms of sex and the BDNF Val66Met interaction, previous research in the general population suggests that female Val/Val carriers tend to exhibit lower cortisol responses to acute stress, such as the Trier Social Stress Test (TSST), whereas male Val/Val carriers demonstrate higher acute cortisol reactivity (68, 69). This indicates that female Val/Val carriers exhibit impaired HPA reactivity. In terms of sex and childhood trauma interaction, previous research in the general population found that females with childhood trauma exhibited blunted cortisol responses to acute stress (TSST) (70), further suggesting impaired HPA reactivity in this group. Furthermore, previous research has shown that HPA reactivity is associated with HCC levels, with healthy individuals with higher HCC exhibiting lower cortisol reactivity to acute stress (67). Therefore, these findings may suggest that female Val/Val carriers with childhood trauma may experience impaired HPA reactivity, which may also have contributed to the elevated HCC levels observed in this study (67). Therefore, a potential shift from resilience via acute reactivity to vulnerability through chronic allostatic loading in the HPA axis (45, 71) may exist in female Val/Val carriers with childhood trauma, which leads to impaired HPA reactivity over time and elevated long-term HPA axis activity (67, 72).

This explanation may be further supported by the potential mechanisms underlying the BDNF Val66Met polymorphism and sex-specific responses to childhood trauma. In Met carriers, reduced hippocampal volume (39, 42), thus impairing the function of the hippocampus in inhibiting the HPA axis (72). This is supported by the prior study that female Met carriers exhibited a stronger cortisol response under acute stress than female Val/Val carriers (68). In contrast, Val/Val carriers exhibit higher BDNF activity, enhancing hippocampal neurogenesis and resilience to neuronal damage (42), which initially supports its function to inhibit the HPA axis under acute stress (73). However, under chronic stress such as childhood trauma, this prolonged inhibiting effect by the hippocampus may lead to exhaustion, impairing regulation over time and resulting in elevated HCC levels according to childhood trauma severity. Furthermore, sex differences amplify this effect. Specifically, women with childhood trauma experience less hippocampal volume reduction than men (74), potentially preserving the normal function of the hippocampus to inhibit the HPA axis (72). In addition, estrogen enhances BDNF expression, which also contributes to the preservation of the hippocampus' function (75, 76). In sum, among female Val/Val carriers, the interplay of preserved hippocampal function and estrogen-enhanced BDNF expression may lead to a more pronounced inhibitory effect of the hippocampus on the HPA axis in response to acute stress. However, prolonged exposure to childhood trauma in these individuals ultimately results in greater systemic exhaustion and significantly elevated HCC levels, serving as a marker of long-term HPA axis dysregulation.

This study had several limitations. First, the nature of the secondary data imposes constraints, as the original study (53) was not specifically designed to address the current research questions. The data included only HCC levels, which represent long-term HPA activity, and did not measure cortisol reactivity to acute stress. Therefore, these results may not provide a complete picture of how cortisol responses are affected by childhood trauma, sex, BDNF polymorphisms, or their interactions. Additionally, the modest sample size may have led to less stable estimates of the associations, underscoring the need for replication with larger samples to confirm these findings. Second, the reliance on subjective self-reported questionnaires, such as the CTQ and PSS, to assess psychological measures introduces potential bias and complicates the analysis of their relationship with HCC, which is an objective physiological measure. Nevertheless, the CTQ and PSS have been reported as reliable tools for assessing childhood trauma history (58) and subjective perception of stress (61), respectively. Third, the sample consisted exclusively of healthy young adults without mental disorders, which may not generalize to other age groups or individuals with mental health conditions. Future studies should address these limitations by including more diverse populations. Fourth, the female-specific three-way interaction effect may reflect estrogen’s enhancement of BDNF expression (76) or greater trauma sensitivity in females (77), although the possibility of random variation cannot be ruled out. Replication of this finding is necessary to confirm its generalizability and explore underlying mechanisms. Finally, confounders may exist and affect the evaluation of HCC levels, as HCC may be influenced by external factors such as hair washing frequency, hair treatment, and BMI (62, 78, 79), which were not fully controlled in this study.

## 5 Conclusion

In summary, the findings suggest a positive relationship between childhood trauma and perceived stress as well as a three-way interaction of childhood trauma × BDNF Val66Met × sex in long-term HPA axis regulation. Specifically, a positive association between childhood trauma and HCC was observed only in female Val/Val carriers, indicating that this subgroup might experience a more pronounced long-term physiological response to childhood trauma. This unique interaction pattern suggests that genetic factors (BDNF Val66Met), environmental factors (childhood trauma), and sex may play crucial roles in shaping chronic physiological responses to stress. Further, these results highlight the public health priority of early trauma intervention and suggest genotype- and sex-specific clinical strategies. Future research should explore the mechanisms underlying these gene- environment interactions and include diverse clinical samples to better understand their implications on mental health.

## Data Availability

The data are not publicly available due to privacy restrictions. Requests to access these datasets should be directed to ansk@yuhs.ac.
